# The Role of Physical Activity in ADHD Management: Diagnostic, Digital and Non-Digital Interventions, and Lifespan Considerations

**DOI:** 10.3390/children12030338

**Published:** 2025-03-07

**Authors:** Alexandra Martín-Rodríguez, Silvia Herrero-Roldán, Vicente Javier Clemente-Suárez

**Affiliations:** 1 Faculty of Medicine, Health and Sports, Universidad Europea de Madrid, Villaviciosa de Odón, 28670 Madrid, Spain; vctxente@yahoo.es; 2 Faculty of Applied Social Sciences and Communications, International Business University (UNIE), 28015 Madrid, Spain; silvia.herrero@universidadunie.com; 3 Grupo de Investigación en Cultura, Educación y Sociedad, Universidad de la Costa, Barranquilla 080002, Colombia

**Keywords:** ADHD, physical activity, exercise, executive function, digital interventions, exergaming, cognitive enhancement, ADHD management

## Abstract

**Background:** Attention Deficit Hyperactivity Disorder (ADHD) has been described as a neurodevelopmental disorder characterized by inattention, hyperactivity, and impulsivity affecting cognitive, emotional, and social functioning. While pharmacological and behavioral treatments remain primary, physical activity (PA) (digital and non-digital versions) has emerged as a great complementary intervention due to its potential impact on executive functions, emotional regulation, and neurobiological markers. **Objectives:** This study aimed to assess the effects of PA on ADHD symptoms, executive function, and emotional regulation, exploring its potential impact and new practical applications in digital and non-digital treatment. **Methods:** This narrative review assessed 132 studies published between 1 January 2010 and January 2025, ensuring the inclusion of the most recent and relevant findings. The review was conducted in Scopus, PubMed, and Web of Science, using a predefined combination of terms related to ADHD, physical activity, executive function, neuroplasticity, and emotional regulation. **Results:** Regular PA improves executive functions, attention, inhibitory control, and cognitive flexibility in ADHD. Aerobic exercise enhances sustained attention, high-intensity training improves impulse control, and coordinative activities boost cognitive flexibility. Non-digital and digital innovations, such as exergaming and wearable fitness trackers, offer promising solutions to improve adherence to PA regimens, reinforcing their role as a key intervention in ADHD management. **Conclusions:** PA could be a valuable complementary intervention for ADHD through a hybrid approach that may improve cognitive and emotional functioning while addressing comorbidities.

## 1. Introduction

Attention Deficit/Hyperactivity Disorder (ADHD) is described as a neurodevelopmental condition that significantly impacts cognitive, emotional, and social functioning [1]. With a global prevalence estimated between 5% and 7% in children and adolescents, and symptoms that often persist into adulthood, ADHD presents a multifaceted challenge in both diagnosis and therapeutic management [2]. Traditional intervention strategies typically have included a combination of pharmacotherapy, psychotherapy, and environmental adjustments in educational and occupational settings. However, in recent years, there has been growing interest in complementary approaches, such as physical activity, due to its potential to alleviate ADHD symptoms and enhance the overall quality of life [3,4].

Consistent physical exercise has demonstrated enhancements in attention, cognitive regulation, and executive functioning in individuals diagnosed with ADHD [5]. Moreover, exercise may enhance neurotransmitters, including dopamine and norepinephrine, which are frequently low in patients with ADHD [5,6]. Physical activity may also improve cerebral blood circulation and cognitive function. Research has shown that physical activity therapies, including aerobic exercise and martial arts, significantly alleviate ADHD symptoms in children and adolescents [7]. Thus, physical activity functions as a potentially beneficial adjunctive treatment for individuals with ADHD [8]. However, individuals with ADHD may encounter various difficulties in engaging in physical activity due to a combination of factors. Many have struggled with motor coordination, which can limit their ability to participate in physical activities effectively. Also, sensory processing challenges are common, making it harder for individuals with ADHD to engage in activities that involve diverse sensory inputs. Additionally, difficulties with executive functioning, which includes skills like planning, organization, and behavioral regulation, can further complicate their ability to participate in activities that require structured effort. These factors contribute to the challenges faced by individuals with ADHD in maintaining consistent active programs. Nevertheless, Zhu et al. have demonstrated the efficacy of physical activity in improving key deficits associated with ADHD, including attention, cognitive control, and executive functioning [7]. Additional research is required to ascertain the most beneficial forms, durations, and intensities of physical exercise therapies for individuals with ADHD. Thus, physical activity must be considered an essential element of a comprehensive ADHD treatment strategy.

This study aimed to assess the effects of PA on ADHD symptoms, executive function, and emotional regulation, exploring its neurophysiological impact and new practical applications in digital and non-digital treatment. The discourse encompasses the impact of physical activity on cognitive functions, including executive functioning and attentional control, as well as its implications on essential neurobiological markers, such as neurotransmitter regulation and brain development. 

In summary, this review synthesizes existing research on physical activity interventions for ADHD, analyzing various approaches such as aerobic exercise, martial arts, and mindfulness-based movement therapies. Special consideration is given to age-specific adaptations to enhance intervention effectiveness. Additionally, the role of physical activity within diagnostic and therapeutic frameworks is explored, emphasizing its potential as a complementary strategy alongside pharmacological and behavioral treatments. Finally, current trends in ADHD-related physical activity research are discussed, providing insights for future applications in clinical and educational settings [9].

### Methodology and Procedures

This review methodology emphasizes the narrative assessment of evidence to provide a balanced and insightful perspective on the relationship between physical activity and ADHD [10]. The search focused on 132 studies published between 1 January 2010 and January 2025, ensuring the inclusion of the most recent and relevant findings. The review was conducted using Scopus, PubMed, and Web of Science, selecting studies published between 2010 and 2025. The search strategy included the following keywords and Boolean operators: “ADHD” OR “Attention Deficit Hyperactivity Disorder” AND “Physical Activity” OR “Exercise” OR “Motor Activity” “Executive Function” OR “Cognitive Flexibility” OR “Inhibitory Control” AND “ADHD” AND “Physical Activity” “Neuroplasticity” OR “Dopamine” OR “Prefrontal Cortex” AND “ADHD” AND “Exercise” “Emotional Regulation” OR “Anxiety” OR “Depression” AND “ADHD” AND “Physical Activity”.

To maintain the quality and focus of this narrative review, the inclusion criteria encompassed peer-reviewed articles focusing on ADHD and physical activity, including meta-analyses, randomized controlled trials (RCTs), and neuroimaging studies assessing the cognitive, emotional, or neurophysiological effects of PA. Studies conducted on children, adolescents, and adults with ADHD were considered, with articles published in English or Spanish between 2010 and 2025. Articles were included in both languages to ensure a diverse and representative sample, as research on this topic has been extensively developed in both linguistic contexts. The inclusion period from 2010 onwards was chosen to focus on recent developments in PA interventions for ADHD, particularly considering the increasing integration of neurophysiological assessments and digital approaches in recent years.

The following exclusion criteria were applied: (i) studies not directly examining the relationship between physical activity and ADHD, (ii) publications with significant methodological limitations or insufficient relevance to the review’s objectives, and (iii) non-peer-reviewed sources, such as conference proceedings, abstracts, or unpublished data. Articles meeting the inclusion criteria were evaluated for their scientific rigor, relevance, and alignment with the review’s subsections. Any discrepancies in article selection or interpretation were resolved collaboratively through group discussions to ensure consensus.

## 2. Core Symptoms and Physical Activity

Research in the field of neuroscience has identified that individuals with ADHD exhibit impairments in regions such as the anterior and posterior cingulate cortex, the prefrontal cortex, the amygdala, the striatum, and the cerebellum [11,12]. This is particularly interesting when considering how the social impairments observed in this population may correlate with a reduced volume of subcortical areas [13].

### 2.1. Core Symptoms and Diagnostic

ADHD is classified as a neurodevelopmental disorder that, although originating in childhood, can persist into adulthood [14]. The Diagnostic and Statistical Manual of Mental Disorders (DSM-5) and the International Classification of Diseases (ICD-11) provide the most widely used diagnostic criteria for ADHD [15,16]. The DSM-5 classifies ADHD into three subtypes:

- Predominantly inattentive presentation, characterized by difficulties in sustaining attention, forgetfulness, and distractibility.
- Predominantly hyperactive-impulsive presentation, marked by excessive movement, restlessness, and impulsive decision-making.
- Combined presentation, where both inattentive and hyperactive-impulsive symptoms are equally present.

An accurate diagnosis is essential for implementing effective treatment strategies, including behavioral therapy, medication, and lifestyle interventions. A misdiagnosis or delayed diagnosis can lead to increased academic failure, occupational difficulties, and mental health issues, reinforcing the importance of a comprehensive and evidence-based approach to ADHD assessment. Most researchers agree on its multifactorial origin, involving both genetic and environmental factors, with the latter potentially operating through epigenetic mechanisms. Genetic studies estimate that heritability rates range between 70 and 80%, with multiple candidate genes associated with dopamine and norepinephrine regulation playing a role in ADHD susceptibility [17]. However, environmental influences, such as prenatal exposure to toxins, low birth weight, maternal stress, and early childhood adversity, may also contribute to the symptom severity [18,19].

Nevertheless, numerous neuroimaging studies aim to explain ADHD from the biological perspective. In this regard, several studies support the potential involvement of the frontostriatal network as a contributing factor to ADHD pathophysiology [14]. Similarly, some investigations suggest that children with ADHD experience a maturational delay in cortical development. For example, in the study conducted by Shaw et al., it was concluded that children with ADHD exhibited greater cortical thinning in key regions for attentional control, providing a possible explanation for the attentional symptoms observed in this sample [20]. However, although originating in childhood, it can persist into adulthood [21]. Most researchers agree on its multifactorial origin, with the latter potentially operating through epigenetic mechanisms [22]. This disorder affects a significant portion of the population, with prevalence estimates indicating approximately 5% in childhood and 2.5% in adulthood. It is characterized by three core symptoms: inattention, impulsivity, and hyperactivity. Inattention primarily affects vigilance, reaction time, and listening ability [17]. In adults, hyperactivity tends to manifest as restlessness, impulsivity, and executive function difficulties, whereas in children it presents as more overt motor hyperactivity. Additionally, emerging perspectives in the literature highlight that emotional regulation deficits observed in these patients should be considered core symptoms of this condition [23,24].

**Figure 1:**
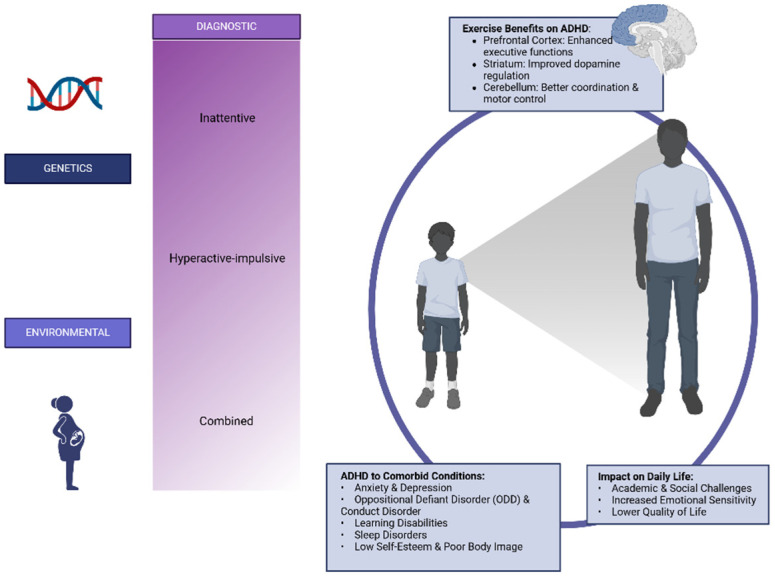
Multifactorial origins, comorbid conditions, impact on daily life, and the role of physical activity and exercise in ADHD management.

Additionally, individuals with ADHD frequently present comorbid disorders. Research indicates a high prevalence of comorbidities with anxiety, depression, oppositional defiant disorder, conduct disorder, and tic disorders [17]. These comorbid disorders can complicate the diagnosis and treatment of ADHD due to frequent symptom overlap. Likewise, ADHD and learning disorders share common characteristics, such as difficulties with attention and memory, posing an additional challenge in academic settings [25]. Additionally, these individuals may experience motor and laterality impairments; weight loss and poorer physical condition; low levels of self-esteem, body image, and self-concept; and sleep problems and disorders [26,27]. All these factors significantly contribute to a lower health-related quality of life for individuals with ADHD [28]. Summarizing the key aspects covered in the following sections, Figure 1 illustrates the multifactorial origins, comorbid conditions, impact on daily life, and the role of physical activity and exercise in ADHD management:

In this regard, the presence of comorbidities has exposed individuals with ADHD to a higher risk of emotional and social problems, increased social and academic maladjustment, and an overall reduction in their ability to function optimally in all aspects of life. Thus, ADHD has gained increasing relevance due to its impact on the social and academic lives of those affected [29].

### 2.2. Physical Activity: An Intervention

The treatment of ADHD has traditionally been based on a complementary pharmacological and psychological approach. The medications of choice in these cases, generally stimulants, have been associated with positive behavioral outcomes. However, approximately 20% of patients do not respond adequately, with some even experiencing a worsening of comorbid disorders [30]. Given this, promising research lines have emerged in the literature focusing on the potential physiological role of physical activity in ADHD symptomatology [31]. Studies on children and adults with normative development highlight the benefits of physical activity on neurocognitive functions that are compromised in individuals with ADHD, suggesting that physical activity could serve as an effective therapeutic complement [32]. For example, research by Muñoz Suazo et al. (2019) has demonstrated the positive effects of physical activity on attention, emphasizing the need to include exercise as a complementary intervention to improve ADHD symptoms, fostering greater interest in non-pharmacological interventions for this population [33]. A recent systematic review and meta-analysis by Seiffer et al. (2022) provided preliminary evidence that moderate-to-vigorous physical activity (MVPA) interventions have small-to-moderate effects on core ADHD symptoms and functional impairment. Additionally, their findings suggest that MVPA offers further benefits for associated impairments, reinforcing its potential as a holistic treatment approach. These results align with growing evidence supporting the integration of structured physical activity as a complementary strategy in ADHD management [34].

However, not all physical activities have the same impact on ADHD symptoms. It would be interesting to mention, now and in the next sections, that recent studies have identified that certain types of exercise may be more beneficial than others.

- Aerobic exercise: Beneficial for improving sustained attention and emotional regulation [35]. A meta-analysis by Yang et al. (2022) found that aerobic exercise significantly improved the executive function in children with ADHD, with moderate-to-large effect sizes for inhibitory control, cognitive flexibility, and working memory. The most effective interventions were moderate-intensity aerobic exercise lasting 60–90 min over 6–12 weeks, with greater benefits observed in children using medication [36].
- High-intensity exercise: Associated with improvements in executive function and inhibitory control [37]. A study by Sun et al. (2022) found a large effect size for the improvement in parent-reported self-monitoring scores in the GameSAE group compared to the control. Additionally, both GameHIIT and GameSAE interventions showed large effects on physical fitness levels compared to the control. However, no significant effects were found for executive function (EF) tests or overall ADHD symptoms, suggesting that a larger intervention dosage may be needed for EF improvements [38].
- Coordinative exercises: These include martial arts and team sports, which promote behavioral control and social interaction [39]. Kadri et al. (2019) found that Taekwondo practice significantly improved cognitive function in adolescents with ADHD, with large effect sizes observed in selective attention and cognitive flexibility. Notably, the Stroop test performance showed strong improvements (ES = 2.16 for color-word interference), and Ruff 2 and 7 test scores indicated enhanced attention control (ES = 2.78 for automated detection). These findings highlight Taekwondo as a promising intervention for ADHD-related cognitive deficits [40].

The inclusion of these personalized approaches in intervention programs could optimize the benefits of exercise for individuals with ADHD.

## 3. Physical Activity’s Impact on Executive Functions

Executive functions refer to a set of cognitive processes classified as highly complex (e.g., planning, decision-making, working memory, emotional and behavioral regulation) that enable the organization, regulation, and execution of goal-directed tasks over the long term [41]. Impairments in these functions have significant implications for an individual’s adaptation, affecting their performance in professional, academic, and social settings. ADHD is also characterized by a range of deficits in these cognitive processes [42].

In this context, the link between physical activity and its effects on the executive functions of individuals with ADHD has been extensively studied. Various meta-analyses and experimental studies suggest that regular physical activity can enhance cognitive functions such as attention, working memory, inhibitory control, cognitive flexibility, and emotional regulation [31,43,44]. These findings align with the hypothesis that physical activity induces neurofunctional and neurostructural changes in the brain, particularly in regions involved in executive functions, such as the frontal lobe and hippocampus, promoting neuroplasticity and improving the efficiency of neural circuits responsible for cognitive and behavioral regulation [45].

Beyond its impact on the frontal lobe and hippocampus, physical activity has been shown to influence other key brain areas implicated in ADHD. The anterior cingulate cortex, which plays a role in attention regulation and impulse control, has been found to exhibit altered activity in individuals with ADHD [46,47]. Regular exercise may help modulate its function, promoting improved attentional control. Additionally, the cerebellum, known for its role in motor coordination, has also been associated with cognitive processes such as task automation and timing regulation [48,49]. Studies suggest that physical activity can enhance cerebellar function, which may contribute to better executive control. Moreover, the default mode network (DMN), a set of brain regions that are typically overactive in ADHD and associated with mind-wandering and distractibility, appears to show greater task-related deactivation following physical activity, supporting enhanced cognitive engagement and self-regulation [49].

Specifically, the meta-analysis by Cerrillo-Urbina et al. (2015) established that physical exercise is effective in reducing symptoms of inattention, impulsivity, hyperactivity, and executive function deficits in children with ADHD [44]. It was also found that physical activity improves processing speed, working memory, planning, and problem-solving in individuals with ADHD. Welsch et al. (2021) also confirmed that physical activity has a positive impact on all executive functions in this population [50]. However, the meta-analysis by Sun et al. (2022) suggested that while physical activity benefits attention, executive functions, and motor skills, there is no clear evidence of improvement in hyperactivity symptoms, indicating the need for additional approaches to address this aspect of the disorder [43].

Furthermore, Liang et al. (2021) reported that physical exercise has moderate-to-large positive effects on inhibitory control and cognitive flexibility, two fundamental components of executive functions [51]. These findings are supported by Xie et al. (2021), who emphasized the efficacy of exercise in improving attention, particularly in reducing inattention symptoms in individuals with ADHD [31], reinforcing the idea that physical activity has a significant impact on cognitive processes essential for adaptation and behavioral regulation. A more recent study by Booth et al. (2024) confirmed these previous findings, indicating that physical activity programs yield significant benefits in inhibition and working memory [52].

These results have been complemented by brain function studies that also highlight the positive effects of physical activity in patients with ADHD. It was found how acute exercise on the electroencephalographic patterns of ADHD patients, drawing two key conclusions. First, individuals with ADHD initially displayed higher theta/beta ratios than control subjects. Second, ADHD individuals who engaged in exercise exhibited lower theta/beta ratios than those who did not, demonstrating that exercise can regulate arousal and alertness levels in individuals with ADHD. Similarly, the study by Hung et al. (2016), using event-related potentials, evidenced that moderate-intensity physical activity has positive effects on the working memory of children with ADHD [8]. Their research showed that ADHD subjects, compared to controls, had longer reaction times, lower accuracy, and greater global cost in task-switching performance. Additionally, ADHD participants exhibited smaller amplitudes and longer P3 latencies in global switch effects. However, after engaging in physical activity, ADHD subjects showed reduced global switch costs and an increased P3 amplitude.

Based on these findings, it can be asserted that the benefits of physical activity extend to executive functions [53], with even more pronounced cognitive benefits in ADHD patients. This can be explained from the pathophysiology of ADHD, as this disorder is associated with inadequate levels of dopamine, serotonin, and norepinephrine [54]. Physical exercise has been shown to increase these three neurotransmitters in the prefrontal cortex, resulting in improvements in attention and executive functions [55].

Regarding neuroimaging studies, the literature has provided substantial evidence that the brains of individuals with ADHD exhibit differences compared to control subjects [56]. ADHD individuals have been reported to have a reduced total brain volume and decreased basal ganglia size (globus pallidus and putamen), as well as increased volume in the left posterior cingulate and precuneus. The authors of [57] suggested that certain regions reduced in childhood in ADHD patients may normalize with age or stimulant medication use, making it plausible that physical activity could exert similar benefits. Concretely, Meijer et al. discovered evidence of substantial beneficial effects of long-term physical activity on neurophysiological functioning, specifying that neurophysiological functioning may be altered by brief physical activity [58].

In this context, physical activity has emerged as a potentially beneficial intervention, not only for treating ADHD symptoms but also for improving cognitive processes that support executive functions, ultimately contributing to better overall functioning in daily life.

## 4. Physical Activity’s Impact on ADHD Comorbidities: Enhancing Emotional Stability, Behavior, and Sleep Patterns

ADHD is a condition that, as previously mentioned, is comorbid with other disorders such as anxiety, depression, behavioral disorders, and sleep disorders, among others. Given this scenario, it is essential that therapeutic interventions not only focus on managing the core symptoms but also integrate the treatment of these comorbidities. Therefore, there is a need for a multidimensional approach that addresses both the associated disorders and ADHD itself, thereby improving the overall effectiveness of treatment.

### 4.1. Emotion and Behavior

In this context, physical activity has been established as a key component for both physical and mental development, with significant benefits in both areas. Numerous scientific studies highlight the effectiveness of exercise in managing disorders such as obesity, hypertension, anxiety, and depression [53]. However, one of the most relevant findings is its positive impact on individuals with ADHD, which justifies its inclusion in the treatment plans for these patients.

From a neuroscientific perspective, various studies have shown that physical activity promotes an increase in brain-derived neurotrophic factor (BDNF) levels, which are reduced in individuals with ADHD. This increase contributes to the improvement of brain function and structure, which in turn enhances the behavior and quality of life of these individuals [59].

Beyond increasing BDNF levels, physical activity has been shown to promote neuroplasticity and structural brain development in individuals with ADHD [59]. Neuroimaging studies suggest that regular exercise contributes to the maturation of the prefrontal cortex, a critical region for attention regulation and executive functioning. Additionally, physical activity fosters improved connectivity within neural circuits involved in motivation and motor regulation, particularly within the basal ganglia, structures that play a key role in ADHD symptomatology. These findings further reinforce the idea that exercise can serve as a long-term neuroprotective intervention for individuals with ADHD.

In terms of mood regulation, scientific evidence has supported the use of physical exercise in improving the emotional and social symptoms associated with ADHD. Research by Cerrillo-Urbina et al. (2015) suggested that physical activity has a positive impact on emotional regulation, helping to reduce depression, anxiety, and stress through increased dopamine levels [44]. More recent studies, such as one by Xie et al. (2021), reinforce the conclusion that physical activity can significantly reduce symptoms of depression and anxiety throughout the lifespan of individuals with ADHD. This, in turn, is related to an increase in social adaptability [31].

Another issue commonly observed in individuals with ADHD, as previously mentioned, is impairment in body image perception, self-esteem, and sleep quality. In this regard, physical activity has also been shown to be beneficial, producing significant improvements in these aspects and thereby enhancing self-esteem as well as sleep quality [51].

While the benefits of physical activity for ADHD are well-documented in childhood and adolescence, it is also important to consider its role in preventing long-term cognitive decline. Emerging research suggests that regular physical activity may help mitigate the risk of neurodegenerative disorders in adults with ADHD by promoting brain plasticity and reducing oxidative stress [27,53]. Given that individuals with ADHD may be at higher risk of executive dysfunction and emotional dysregulation later in life, incorporating physical exercise into daily routines could serve as a proactive strategy to maintain cognitive health and overall well-being. Thus, to maximize the therapeutic impact of physical activity, it is essential to consider its integration with other established ADHD interventions. Studies indicate that combining exercise with cognitive-behavioral therapy (CBT) or mindfulness-based strategies can enhance treatment adherence and boost outcomes in reducing impulsivity and emotional instability [60]. The physiological benefits of exercise, such as [61] increased dopamine and serotonin levels, complement psychological interventions by improving mood regulation and attentional control. This integrative approach underscores the importance of physical activity as a key element in a multidimensional treatment plan for ADHD.

### 4.2. Sleep Patterns

Sleep disturbances are commonly reported in individuals with ADHD, with issues such as difficulty falling asleep, reduced sleep duration, frequent nighttime awakenings, and lower sleep quality significantly impacting daily functioning. Research has shown that these sleep problems contribute to worsening core ADHD symptoms, including inattention, impulsiveness, and emotional dysregulation, further complicating academic, social, and occupational performance [62].

Physical activity has been recognized as an effective, non-pharmacological intervention for improving sleep patterns in individuals with ADHD [63]. Engaging in regular exercise has been associated with increased sleep efficiency, reduced sleep onset latency, and improved overall sleep quality. This improvement is largely attributed to the role of physical activity in regulating the hypothalamic–pituitary–adrenal (HPA) axis, which helps modulate stress responses and circadian rhythms, both of which are often dysregulated in individuals with ADHD [63].

Moreover, exercise contributes to sleep regulation through its effects on neurotransmitter activity. Alnawwar et al. suggest that physical activity promotes the release of serotonin and melatonin, both of which are crucial for initiating and maintaining restful sleep [64]. Additionally, engaging in moderate-intensity aerobic exercise has been found to reduce hyperarousal, a common issue in individuals with ADHD that can lead to restlessness and difficulty in achieving deep sleep stages [65].

The type and timing of physical activity are also relevant factors. While moderate-intensity aerobic and resistance exercises have been found to improve sleep quality, engaging in high-intensity physical activity too close to bedtime may lead to increased physiological arousal, potentially delaying sleep onset [66]. Therefore, incorporating structured exercise routines earlier in the day may be the most effective approach to optimizing sleep benefits for individuals with ADHD.

As mentioned by Mulraney et al., the bidirectional relationship between ADHD symptoms and sleep disturbances should be considered [67], and incorporating physical activity as part of a comprehensive treatment plan can be an essential strategy to mitigate both issues. Improved sleep quality, in turn, enhances cognitive functioning, emotional regulation, and overall well-being, further reinforcing the role of exercise as a crucial therapeutic tool for individuals with ADHD [67].

In summary, physical activity emerges as a comprehensive therapeutic tool that not only aids in managing the core symptoms of ADHD but also positively impacts associated comorbidities such as anxiety, depression, and sleep disorders. Therefore, the incorporation of physical exercise programs should be considered an essential complementary intervention in the treatment of ADHD.

## 5. Recent Physical Activity Types and Interventions

**Figure 2:**
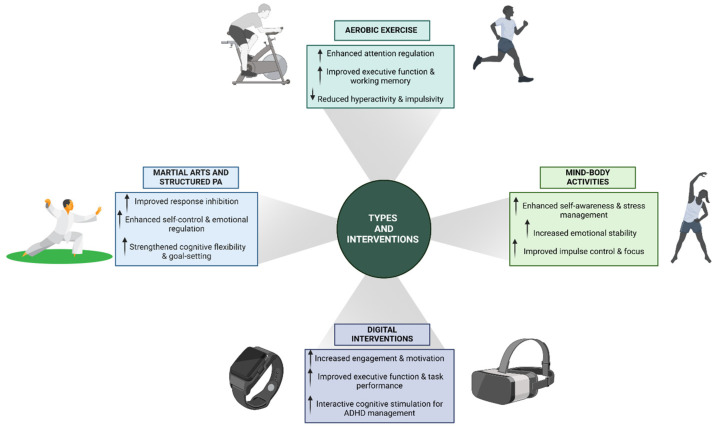
Types and interventions.

In recent years, there has been a growing interest in the role of PA as a complementary intervention for managing ADHD. Various forms of PA have been explored to determine their efficacy in alleviating core symptoms and enhancing cognitive functions in individuals with ADHD. This section reviews recent studies focusing on different types of PA interventions (Figure 2) and their impacts on ADHD symptoms.

### 5.1. Aerobic Exercise Interventions for ADHD Management

Aerobic exercise has been widely studied as a non-pharmacological intervention for individuals with ADHD, showing promising results in alleviating core symptoms such as inattention, hyperactivity, and impulsivity [35]. Engaging in activities such as running, cycling, and swimming has been found to significantly enhance executive function, working memory, and response inhibition in individuals with ADHD [44]. The benefits of aerobic exercise stem from its ability to modulate neurobiological processes, improve cardiorespiratory fitness, and induce favorable neurochemical changes that positively influence cognitive and behavioral outcomes [59]. This section explores the mechanisms through which aerobic exercise impacts ADHD symptoms and reviews the most recent evidence supporting its efficacy.

Aerobic exercise influences ADHD symptomatology through multiple neurobiological pathways, primarily by enhancing dopaminergic and noradrenergic neurotransmission, which are known to be dysregulated in individuals with ADHD [68]. Studies using neuroimaging techniques have demonstrated that aerobic activity increases dopamine and norepinephrine levels in the prefrontal cortex, areas crucial for attention, impulse control, and cognitive flexibility [69]. Additionally, aerobic exercise has been linked to increased brain-derived neurotrophic factor (BDNF), a protein essential for neuroplasticity, synaptic function, and overall brain health [70].

A randomized controlled trial by Pan et al. (2016) assessed the effects of a 12-week aerobic exercise program on children with ADHD and found significant improvements in sustained attention and cognitive flexibility, attributed to enhanced frontal lobe activity and increased neurotrophic factor levels [71]. Moreover, aerobic exercise has been associated with greater white matter integrity in regions linked to executive functioning, which may contribute to long-term cognitive benefits in individuals with ADHD [72].

Several studies have demonstrated that structured aerobic exercise programs can lead to improvements in ADHD-related symptoms. A meta-analysis conducted by Cerrillo-Urbina et al. (2015) analyzed multiple randomized controlled trials and concluded that aerobic exercise significantly enhances selective attention, working memory, and inhibitory control in children and adolescents with ADHD. The improvements in cognitive function were particularly pronounced when exercise was performed regularly for at least 30–45 min per session, three to five times per week [44].

Furthermore, a study by Pontifex et al. (2013) found that a single bout of moderate-intensity aerobic exercise improved task performance and neural efficiency in children with ADHD, as measured using event-related potentials (ERPs) during cognitive testing [73]. The authors suggested that aerobic exercise may serve as a short-term strategy to enhance cognitive performance before engaging in academic tasks. Similarly, a study by Choi et al. (2014) reported that adolescents with ADHD who participated in a six-week treadmill running program exhibited significant reductions in hyperactivity and impulsivity, as measured using behavioral assessments and teacher reports [35,70].

Beyond immediate cognitive benefits, regular aerobic exercise has been linked to long-term improvements in ADHD symptoms and overall mental health. A longitudinal study examined the effects of sustained aerobic activity on children with ADHD and found that participants who maintained a physically active lifestyle over a one-year period showed continued enhancements in attention span, emotional regulation, and academic performance [74]. The authors emphasized the importance of consistency and adherence to exercise programs in maximizing benefits for ADHD symptomatology.

Additionally, aerobic exercise has been associated with reduced reliance on stimulant medications for ADHD management. A study by Smith et al. found that children with ADHD who engaged in regular aerobic activity required lower doses of methylphenidate to achieve symptom control compared to their sedentary peers. This suggests that integrating structured aerobic exercise into ADHD treatment plans may reduce the need for high pharmacological doses and mitigate potential side effects associated with stimulant medications [75].

To optimize the effectiveness of aerobic exercise interventions for ADHD, specific program design elements should be considered. Research suggests that exercise intensity, duration, and frequency play critical roles in determining cognitive and behavioral outcomes. A systematic review by Verret et al. (2010) recommended the following guidelines for aerobic exercise interventions in ADHD populations [3]:

- Duration: Sessions should last at least 30–45 min to produce noticeable cognitive benefits.
- Frequency: A minimum of three to five sessions per week is recommended to sustain improvements.
- Intensity: Moderate-to-vigorous intensity (60–80% of maximum heart rate) has been shown to yield optimal outcomes.
- Engagement: Activities should be enjoyable and structured to promote adherence and motivation.

Moreover, incorporating social and competitive elements into aerobic exercise programs may enhance engagement in children with ADHD. Activities such as team sports, group cycling, and supervised running programs have been reported to improve adherence and provide additional social benefits, such as enhanced peer interactions and teamwork skills [76].

Aerobic exercise has emerged as a highly effective, non-pharmacological intervention for ADHD management, with significant benefits for cognitive function, attention regulation, and behavioral control. The underlying neurobiological mechanisms, including enhanced dopamine transmission and increased BDNF levels, contribute to the positive impact of aerobic exercise on ADHD symptoms. Research strongly supports the integration of structured aerobic activities into treatment plans to complement traditional pharmacological and behavioral therapies. Future studies should focus on optimizing exercise protocols, exploring long-term adherence strategies, and identifying individualized approaches to maximize benefits for different ADHD subtypes.

Finally, mindfulness-based physical activities, such as yoga, have gained increasing recognition for their potential to enhance behavioral and cognitive functions in individuals with ADHD. These activities combine controlled movement, breathing exercises, and mindfulness practices, which may help improve self-regulation and attentional control. A randomized controlled trial by Cohen et al. (2018) demonstrated that yoga interventions led to significant reductions in hyperactivity and impulsivity, as well as improvements in attention in children with ADHD [77,78]. Similarly, research by Hariprasad et al. (2013) found that regular yoga practice enhanced working memory and executive functioning, suggesting its viability as a complementary therapeutic approach. The incorporation of mindfulness elements in physical activity may help individuals with ADHD develop improved emotional regulation, stress management, and cognitive flexibility, making yoga a valuable addition to ADHD treatment plans [78].

### 5.2. Martial Arts and Structured Physical Activity

Structured physical activities, particularly martial arts, have garnered attention as potential non-pharmacological interventions for managing ADHD. These disciplines combine physical exertion with mental focus, discipline, and self-regulation, which may address core ADHD symptoms such as inattention, impulsivity, and hyperactivity. This section explores recent research on the impact of martial arts and other structured physical activities on individuals with ADHD.

Martial arts training emphasizes concentration, discipline, and the execution of precise movements, which can enhance cognitive functions. A study by Lakes and Hoyt (2004) investigated the effects of a traditional martial arts program on children aged 8 to 11 and found significant improvements in self-regulation, attention, and classroom behavior among participants. The structured nature of martial arts, emphasizing self-control and focus, may contribute to these positive outcomes, making it a promising intervention for children with ADHD [79].

However, not all studies have found positive effects. A study by Ludyga et al. (2022) investigated the behavioral and neurocognitive effects of judo training on response inhibition in children with ADHD and children born very preterm [74]. The results indicated that while judo training improved response inhibition in children born very preterm, it did not elicit the same benefits in children with ADHD. The authors suggested that the complexity of judo might have posed excessive demands on children with ADHD, potentially hindering the refinement of techniques and cognitive benefits.

Taekwondo, a Korean martial art known for its dynamic kicks and emphasis on discipline, has also been studied for its effects on cognitive functions in adolescents with ADHD. A study by Kadri et al. (2019) examined the impact of a Taekwondo program on selective attention in adolescents with ADHD [80]. The findings revealed significant improvements in selective attention, as measured using the Ruff 2 and 7 Selective Attention Test, suggesting that Taekwondo practice may enhance attentional control in this population.

The potential benefits of martial arts and structured physical activities for individuals with ADHD may be attributed to several mechanisms. Firstly, the physical exertion involved in these activities can lead to increased levels of neurotransmitters, such as dopamine and norepinephrine, which play crucial roles in attention and executive function. Secondly, the structured environment and repetitive practice inherent in martial arts may promote the development of self-discipline, goal-setting, and delayed gratification, skills often deficient in individuals with ADHD. Lastly, the mindfulness and meditative aspects of martial arts training can enhance self-awareness and emotional regulation, further contributing to improved cognitive and behavioral outcomes [81].

While research on martial arts and structured physical activities as interventions for ADHD is still emerging, initial findings suggest potential benefits in improving attention, self-regulation, and behavior [82,83]. However, the variability in outcomes across different studies indicates the need for further research to identify the specific components of these activities that are most effective and to determine the optimal frequency, intensity, and duration of interventions. Tailoring programs to the individual needs and capabilities of participants with ADHD will be essential to maximize the therapeutic potential of these structured physical activities.

### 5.3. Emerging Trends in Physical Activity Research for ADHD

The exploration of physical activity as a therapeutic intervention ADHD has gained momentum in recent years. While current evidence supports the benefits of PA in mitigating ADHD symptoms, ongoing research continues to uncover new insights and innovative approaches. This section delves into emerging trends in PA research for ADHD, highlighting novel interventions, technological integrations, and personalized strategies aimed at enhancing treatment efficacy.

Although we will explore this topic in a separate section, we could not overlook the digital applications of current and future interventions in this one. The fusion of technology with PA interventions presents a promising avenue for ADHD management. Exergaming, which combines exercise with interactive video games, has emerged as an engaging method to promote physical activity among individuals with ADHD. A study by Staiano et al. (2017) demonstrated that an active video game intervention led to improvements in executive functions and a reduction in inattentive behaviors in children with ADHD [84]. Similarly, virtual reality (VR)-based exercises offer immersive environments that can be tailored to individual needs, providing both physical engagement and cognitive challenges. These technological integrations not only make PA more appealing but also allow for the customization of interventions to target specific ADHD symptoms.

Recognizing the heterogeneity of ADHD presentations, there is a growing emphasis on personalized PA programs. Tailoring interventions to individual preferences, fitness levels, and symptom profiles may enhance adherence and therapeutic outcomes. A systematic review by Cerrillo-Urbina et al. (2015) highlighted the variability in responses to different types of exercise among individuals with ADHD, suggesting that personalized approaches could optimize benefits. Future research is focusing on identifying biomarkers and utilizing machine learning algorithms to predict individual responses to various PA interventions, thereby facilitating the development of customized treatment plans [44].

While short-term benefits of PA on ADHD symptoms are well-documented, there is a need for longitudinal studies examining the long-term effects of sustained physical activity. Understanding how continuous engagement in PA influences the developmental trajectory of ADHD symptoms and associated cognitive functions is crucial. It was shown how children with ADHD who maintained regular physical activity over a year exhibited sustained improvements in attention and executive functions [39]. Such findings underscore the importance of long-term adherence to PA and the necessity for further research in this domain.

Mind–body interventions, which combine physical activity with mindfulness or meditative practices, are gaining attention in ADHD research. Activities such as yoga and tai chi incorporate elements of physical exertion, breath control, and mental focus, potentially addressing both the physical and cognitive aspects of ADHD. A randomized controlled trial by Cohen et al. (2018) found that a yoga intervention led to significant reductions in hyperactivity and impulsivity in boys with ADHD. Future studies are exploring the mechanisms underlying these effects and the potential for integrating mind–body interventions into comprehensive ADHD treatment plans [77].

Advancements in neuroimaging and neurophysiological assessment tools are enabling researchers to delve deeper into the neurobiological mechanisms through which PA exerts its effects on ADHD symptoms. Studies utilizing functional magnetic resonance imaging and electroencephalography examine changes in brain activity patterns pre- and post-exercise interventions. For instance, research by Choi et al. (2014) demonstrated alterations in neural activation associated with improved inhibitory control following an aerobic exercise program in adolescents with ADHD [35]. Understanding these mechanisms can inform the optimization of PA interventions and the identification of individuals who may benefit the most.

## 6. Age-Specific Considerations: Designing Interventions for Maximum Impact

When developing physical activity interventions for individuals with ADHD, it is crucial to tailor programs to the specific developmental stages of participants. Age-specific considerations ensure that interventions are developmentally appropriate, engaging, and effective in addressing the unique challenges associated with ADHD across the lifespan.

### 6.1. Children (6–12 Years)

For children aged 6 to 12 years diagnosed with ADHD, PA interventions can play a pivotal role in managing symptoms and enhancing overall well-being. This developmental stage is characterized by significant cognitive, motor, and social growth, making it an opportune time to integrate structured PA programs tailored to the unique needs of this population.

Engaging in regular PA has been shown to alleviate core ADHD symptoms, including inattention, hyperactivity, and impulsivity. A systematic review by Cerrillo et al. (2015) concluded that PA interventions led to significant improvements in these areas among children with ADHD. The mechanisms underlying these benefits are thought to involve enhanced dopaminergic and noradrenergic activity in the brain, which are critical for attention and executive functioning [44].

Executive functions, such as working memory, cognitive flexibility, and inhibitory control, are often impaired in children with ADHD. PA has been found to bolster these cognitive processes. A meta-analysis by Tan et al. (2022) reported that PA interventions yielded moderate-to-large positive effects on overall executive functioning in this demographic. Activities that require strategic planning and decision-making, such as team sports, may be particularly effective in this regard [85].

Beyond cognitive enhancements, PA provides social and emotional advantages. Participating in group activities fosters teamwork, communication skills, and peer relationships, which can be challenging for children with ADHD. Moreover, regular PA has been associated with reductions in anxiety and depression symptoms, contributing to improved emotional regulation.

To maximize the benefits of PA for children with ADHD, interventions should have the following characteristics:

- Enjoyable and engaging: Selecting activities that align with the child’s interests increases motivation and adherence.
- Structured yet flexible: While structure provides predictability, allowing some flexibility accommodates individual differences and prevents frustration.
- Developmentally appropriate: Activities should match the child’s developmental level to ensure they are challenging yet achievable.
- Incorporate cognitive challenges: Games that require problem-solving and strategy can enhance executive functions.

Active involvement from parents and educators is crucial. They can provide support, encouragement, and reinforcement, creating an environment that values and promotes physical activity. Collaborating with professionals to monitor progress and adjust programs as needed ensures that interventions remain effective and responsive to the child’s evolving needs. Implementing tailored PA interventions for children aged 6 to 12 with ADHD offers a multifaceted approach to symptom management. By addressing cognitive, social, and emotional domains, these programs can significantly enhance the quality of life and developmental trajectory of affected children.

### 6.2. Adolescents (13–18 Years)

Adolescence is a critical developmental period marked by significant physical, cognitive, and emotional changes. For individuals aged 13 to 18 years diagnosed with ADHD, tailored physical activity interventions can serve as effective non-pharmacological strategies to manage symptoms and promote overall well-being.

Regular engagement in PA has been associated with reductions in core ADHD symptoms, including inattention, hyperactivity, and impulsivity. A systematic review by Xie et al. (2021) concluded that PA interventions significantly improved attention-related symptoms in individuals with ADHD [31]. The meta-analysis indicated that both open motor skills (e.g., team sports) and closed motor skills (e.g., structured exercises) were beneficial, with open motor skills showing a more substantial impact on attention problems.

Adolescence is a pivotal time for the development of executive functions, which are often impaired in individuals with ADHD. Physical activity has been shown to enhance these cognitive processes. It was highlighted that PA interventions had a positive effect on executive functions in children with ADHD, suggesting potential benefits for adolescents as well [50].

Participating in group-based PA, such as team sports, offers adolescents opportunities to develop social skills, including teamwork, communication, and leadership. These experiences can enhance self-esteem and provide a sense of belonging, which are particularly beneficial for adolescents with ADHD who may struggle with social interactions. Moreover, regular PA has been linked to improvements in mood and reductions in anxiety and depression symptoms, contributing to better emotional regulation [86].

To maximize the benefits of PA interventions for adolescents with ADHD, consider the following:

- Activity selection: Incorporate a variety of activities that combine aerobic and skill-based components to maintain engagement and address different aspects of physical and cognitive development.
- Structure and routine: Establish consistent schedules and clear expectations to provide stability, which can help adolescents with ADHD manage time and responsibilities effectively.
- Goal setting: Encourage adolescents to set personal goals related to PA, fostering a sense of ownership and motivation.
- Parental and peer support: Involve parents and peers to create a supportive environment that reinforces positive behaviors and adherence to the PA program.

Implementing structured PA interventions during adolescence can play a crucial role in managing ADHD symptoms and supporting overall development. By addressing cognitive, social, and emotional domains, these programs can enhance the quality of life for adolescents with ADHD and promote lifelong healthy habits.

### 6.3. Adults (19 Years and Older)

For adults aged 19 and older diagnosed with ADHD, incorporating regular PA can serve as a valuable non-pharmacological strategy to manage symptoms and enhance overall quality of life. While pharmacological treatments remain a primary approach, PA offers complementary benefits that address both core ADHD symptoms and associated comorbidities.

Engaging in regular PA has been associated with reductions in core ADHD symptoms, including inattention, hyperactivity, and impulsivity. A study by Mehren et al. (2019) reviewed the effects of exercise on ADHD symptoms and concluded that PA could serve as a beneficial adjunct treatment for adults with ADHD. The authors noted that exercise might enhance executive functioning and attention, contributing to improved daily functioning [87].

Executive functions, such as working memory, cognitive flexibility, and inhibitory control, are often impaired in adults with ADHD. Regular aerobic exercise has been shown to improve these cognitive processes. A study by Gapin et al. (2015) found that adults with ADHD who participated in an aerobic exercise program demonstrated significant improvements in executive functioning, particularly in areas related to attention and planning [88]. These findings suggest that PA can play a crucial role in mitigating cognitive deficits associated with ADHD [89].

Adults with ADHD frequently experience comorbid conditions such as anxiety and depression. Regular PA has been shown to alleviate symptoms of these conditions by reducing stress and enhancing mood through the release of endorphins and other neurochemicals. A review by Ashdown-Franks et al. (2019) highlighted that exercise interventions could lead to significant reductions in depressive symptoms among adults with ADHD, thereby improving overall mental health [90].

To maximize the benefits of PA interventions for adults with ADHD, consider the following:

- Personalization: Tailor activities to individual preferences to enhance motivation and adherence.
- Structure and routine: Establish consistent schedules to provide predictability, which can aid in time management and reduce procrastination.
- Social support: Encourage participation in group activities or exercise with a partner to increase accountability and provide social interaction.
- Goal setting: Set realistic and achievable goals to foster a sense of accomplishment and maintain engagement.

Incorporating regular physical activity into the lifestyle of adults with ADHD offers a multifaceted approach to managing symptoms and improving overall well-being. By addressing cognitive deficits, enhancing mental health, and promoting physical fitness, PA serves as a valuable adjunct to traditional treatment modalities for adult ADHD [91].

## 7. Digital Interventions Based on PA and Therapeutic Integration

Digital methods have surfaced as effective instruments for tackling the cognitive and socioemotional difficulties encountered by patients with ADHD. Diverse technologies, such as video games, mobile applications, virtual and augmented reality, and neurofeedback, have been utilized to assist in both diagnosis and treatment. Recent data indicate that serious games can significantly improve executive functions, including working memory, attention, cognitive flexibility, and metacognitive and emotional control skills [92]. By incorporating various digital technologies, these interventions provide a tailored and interactive method for managing ADHD, underscoring their applicability in therapeutic and educational environments. Additionally, technology offers a more dynamic and objective assessment by providing real-time data on PA patterns and their impact on ADHD symptoms. In addition to diagnosis, a multimodal therapeutic approach that integrates cognitive-behavioral therapy (CBT), medication, and PA is garnering recognition for its synergistic effects [93].

### 7.1. Wearable Technology

Innovative diagnostic tools are transforming the assessment and management of ADHD, particularly through the use of wearable devices and behavioral tracking. These technologies enable real-time monitoring of physical activity (PA) patterns and their potential impact on ADHD symptoms, providing objective data that complement traditional diagnostic methods [94]. Denyer et al. specified that remote measurement technology (RMT) offers the capacity to tackle existing research and clinical obstacles associated with ADHD symptoms and its concomitant mental health issues [93]. Although research utilizing RMT has been effectively implemented in other populations, adherence and attrition present significant challenges when applying RMT to a disease like ADHD [94]. Nonetheless, wearable technology utilization among children and adolescents demonstrated feasibility and efficacy in health promotion. Zhang et al. reported a systematic review that synthesized current research on the application of wearable technology in enhancing health among various young demographics, encompassing both healthy and sick persons [95].

### 7.2. Digital Interventions Based on Playing

In this regard, digital interventions have emerged as promising tools for addressing the cognitive and socioemotional challenges faced by children with ADHD [94]. Various technologies, including video games [96], mobile applications, virtual and augmented reality, and neurofeedback, have been employed to support both diagnosis and treatment [97]. Recent evidence suggests that serious games can effectively enhance executive functions such as working memory, attention, and cognitive flexibility, as well as metacognitive and emotional regulation skills [98]. A randomized controlled trial carried out by Wilkes-Gillan et al. examined the effectiveness of a play-based intervention in improving social skills in children with ADHD, showing significant peer interaction improvements and sustained long-term effects [99]. However, a cross-sectional study conducted in Canada examined video game use in children with ADHD, revealing higher addiction scores and prolonged playtime compared to non-ADHD peers, with ADHD severity correlating significantly with excessive gaming [100]. Despite this, a systematic review analyzed the effectiveness of video game-based tools for assessing and managing ADHD in children, finding that they can successfully differentiate ADHD cases from controls and improve cognitive functions and symptom management. Regarding PA, recent studies indicate that combining digital therapies with physical activity-based techniques may enhance their efficacy in managing ADHD. Exergaming, which merges physical activity with digital gaming, has shown promise in improving executive function and impulse control by promoting active participation and strengthening cognitive abilities [101]. Utilizing physical activity inside digital platforms, these therapies deliver cognitive stimulation while simultaneously mitigating hyperactivity and impulsivity through organized movement, thus presenting a holistic and engaging therapy strategy for ADHD management [102]. Thus, by integrating multiple digital solutions, these interventions offer a personalized and engaging approach to ADHD management, highlighting their potential for inclusion in therapeutic and educational settings. However, the Kim et al. study warned about protocols on mobile game-based digital interventions for children with ADHD and analyzed the impact of perceived difficulty and enjoyment on performance, finding that therapeutic goals should take precedence over flashy visuals or difficulty adjustments to enhance engagement [103].

### 7.3. Artificial Intelligence and Machine Learning in ADHD Management

Artificial intelligence (AI) and machine learning (ML) are increasingly being incorporated into ADHD diagnosis and treatment, providing predictive insights and optimizing personalized intervention strategies. Machine learning is a branch of artificial intelligence that utilizes algorithms to enable computers to discern patterns in extensive datasets and perform predictive analysis [104]. This learning enables computers to execute specified tasks independently, without requiring programming. The word was initially employed in 1959. Nonetheless, it has attained significance in recent years owing to the surge in computational capacity and the proliferation of data. Machine learning methodologies are, indeed, an essential component of Big Data [105]. ML algorithms have been employed to refine ADHD diagnosis by identifying biomarkers and classifying symptom severity based on real-time data collection.

In therapeutic settings, AI-enhanced adaptive learning platforms may tailor interventions to an individual’s cognitive profile, adjusting difficulty levels and feedback mechanisms dynamically to enhance engagement and effectiveness [106]. Furthermore, AI-based chatbots and virtual assistants are being explored for providing immediate support in managing ADHD-related challenges, such as organization, time management, and emotional regulation [107]. Recent research has also begun to explore the intersection between AI and PA in ADHD management [108]. AI-driven exercise tracking systems, and motion analysis tools can provide real-time feedback on movement patterns, motor coordination, and energy expenditure, helping individuals with ADHD engage in structured PA programs tailored to their needs. Moreover, a scoping review led by An et al. pointed out that AI-powered virtual coaching platforms use machine learning to analyze behavioral patterns and suggest personalized PA interventions that optimize cognitive and emotional benefits [109].

In summary, by leveraging large datasets and predictive analytics, these technologies can enhance diagnostic accuracy, optimize treatment plans, and provide continuous support for individuals with ADHD. However, challenges, such as ethical considerations, data privacy, and accessibility, must be addressed to ensure equitable implementation.

### 7.4. Virtual Reality and Augmented Reality Applications

Immersive technologies, such as virtual reality (VR) and augmented reality (AR), are proving to be powerful tools in ADHD intervention by creating controlled and interactive environments that enhance cognitive training [110]. VR-based therapies provide simulations that mimic real-life scenarios, helping individuals develop executive functioning skills like impulse control, sustained attention, and emotional regulation in a safe and engaging manner [111]. AR applications, on the other hand, blend digital elements with real-world tasks to promote structured learning experiences, improve task engagement, and reinforce positive behaviors. These technologies have demonstrated potential in both educational and clinical settings, offering engaging ways to enhance focus and self-regulation in children with ADHD [112,113]. A randomized experimental study carried out by Cunha et al. evaluated the effectiveness of a virtual reality-based intervention in students with ADHD symptoms, finding significant improvements in processing speed but limited effects on working memory, suggesting potential benefits for cognitive training [114].

In this regard, PA has been shown to increase dopamine and norepinephrine levels, which could also be targeted using VR and AR training [115], potentially leading to synergistic benefits when combined. Incorporating movement-based strategies, such as exergaming and virtual reality-assisted physical exercise, can provide a dynamic and engaging method to reinforce self-regulation and executive function improvements. Studies indicate that active participation in PA, coupled with real-time neural feedback, may further optimize cognitive flexibility, motor control, and impulse regulation, creating a more comprehensive and holistic intervention approach for ADHD management [116]. Although the ability of VR and AR to simulate real-world challenges in a controlled setting makes them valuable tools for skill development and behavioral reinforcement, further research is needed to refine these interventions, optimize their long-term efficacy, and ensure accessibility for diverse populations [117].

### 7.5. Neurofeedback and Brain–Computer Interfaces (BCIs)

Neurofeedback is an emerging non-pharmacological intervention that utilizes real-time monitoring of brain activity to improve self-regulation in individuals with ADHD [118]. Electroencephalography (EEG)-based neurofeedback trains patients to modify their brain wave patterns, thereby enhancing attention and reducing impulsivity and hyperactivity symptoms [118]. Advances in brain–computer interfaces (BCIs) are also being explored to provide direct communication between the brain and digital devices, allowing individuals with ADHD to control technology using neural signals [119]. While still in the early stages, BCIs hold promise for personalized intervention strategies, enabling real-time adjustments based on neural activity and offering novel approaches for cognitive enhancement and behavioral modification. Also, a recent study reinforced their potential for improving attention, working memory, and socio-emotional skills in students with neurodevelopmental disorders, highlighting their role in personalized training and rehabilitation [120]. However, a study on electronic brain–computer interfaces (eBCIs) highlights not only their practical implications but also deeper ethical and philosophical questions about selfhood, identity, and human augmentation [119].

The combination of PA with neurofeedback training represents an innovative, multimodal approach that aligns with ADHD’s neurobiological characteristics, addressing both cognitive and behavioral symptoms. Research suggests that incorporating PA into BCI interventions could leverage the benefits of exercise-induced neuroplasticity, which enhances cognitive flexibility, motor coordination, and impulse control [102,121].

As neurofeedback and BCIs continue to advance, their potential for ADHD intervention and cognitive enhancement grows, offering real-time, personalized strategies for improving attention and self-regulation. However, alongside their therapeutic promise, these technologies raise important ethical and philosophical concerns about identity, autonomy, and the evolving nature of human cognition, necessitating further interdisciplinary research and ethical scrutiny.

## 8. Future Directions: Engagement in Physical Activity Research for ADHD

As research continues to uncover the intricate relationship between physical activity (PA) and ADHD, several emerging trends are shaping future investigations and interventions. These trends range from digital to non-digital approaches, encompassing technological advancements, personalized PA programs, long-term adherence strategies, sports, and neurobiological assessments. As Liu et al. pointed out, the digital interventions proved beneficial for individuals with ADHD by alleviating symptoms of ADHD, inattention, and hyperactivity/impulsivity [122]; however, screen dependency may increase impulsivity or overstimulation. In contrast, more traditional approaches reduce hyperactivity in a physical way and enhance social skills and emotional regulation, but require motivation and consistency.

In this regard, recognizing the heterogeneity of ADHD presentations, there is a growing emphasis on individualized PA programs. Tailoring interventions based on personal preferences, fitness levels, and symptom profiles may enhance adherence and therapeutic outcomes. Current research is exploring biomarkers and utilizing machine learning algorithms to predict individual responses to various PA interventions, thereby facilitating the development of customized treatment plans. The focus on precision medicine in ADHD treatment could extend to PA, where interventions are specifically designed to optimize benefits for different ADHD subtypes.

### Studies on Sustained PA Engagement and Improvements with ADHD Symptoms: From Non-Digital to Digital

Studies that follow individuals across developmental stages, from childhood to adulthood, using digital or non-digital interventions, could provide insights into the lasting impact of PA on cognitive and behavioral outcomes, as well as its role in reducing reliance on pharmacological treatments [123]. Table 1 serves to highlight a subset of studies that specifically examine the long-term impact of sustained physical activity interventions on ADHD symptoms. These studies offer a mix of digital and non-digital interventions, allowing for a comparison of traditional exercise-based approaches with emerging technology-driven methods [124]. The selection criteria focused on the studies that carried out the following:

- Evaluated the impact of sustained physical activity interventions over weeks.
- Assessed cognitive, behavioral, or physiological outcomes relevant to ADHD.
- Represented both structured and adaptable intervention formats.

In summary, the best ADHD intervention depends on individual needs, combining digital and non-digital approaches for optimal results. Digital tools, like exergaming, VR, and mobile apps, enhance engagement, cognitive training, and self-monitoring, while non-digital activities, such as aerobic exercise, martial arts, and mindfulness, improve dopamine regulation, executive function, and emotional control. A hybrid model—using digital tools to support physical activity—can maximize benefits. Ultimately, any intervention is effective if it leads to positive outcomes, emphasizing consistency and personalization in ADHD management.

## 9. Summary of Practical Applications

The growing body of research on physical activity (PA) and its impact on ADHD management underscores the need for practical, evidence-based applications in clinical, educational, and everyday settings. Advancements in neuroimaging and neurophysiological assessments are enabling researchers to explore the underlying mechanisms through which PA exerts its effects on ADHD symptoms [54]. Functional magnetic resonance imaging (fMRI) and electroencephalography (EEG) studies are examining brain activity patterns before and after PA interventions, helping to identify specific neural circuits associated with cognitive improvements. By elucidating these neurobiological pathways, researchers can optimize PA interventions and identify individuals who may benefit most from specific types of exercise [125,131,132,133,134]. Given the accumulating evidence supporting PA’s role in ADHD management, policymakers and educators must consider integrating structured PA programs into school and clinical settings.

Based on the findings reviewed in this paper, several key takeaways can be applied to optimize ADHD symptom management through structured PA interventions:

### 9.1. Implementing Physical Activity in Clinical and Educational Contexts

- Clinical Settings:
  o Healthcare providers should incorporate PA recommendations as part of multimodal ADHD treatment plans, alongside pharmacological and behavioral interventions [126].
  o Structured PA programs, such as aerobic exercise, strength training, and mindfulness-based movement activities (e.g., yoga, tai chi), should be prescribed based on patient needs and preferences.
  o Clinicians should monitor adherence and effectiveness through wearable technology and digital tracking tools to provide personalized feedback.
- Educational Settings:
  o Schools should integrate structured exercise breaks, active learning strategies, and after-school sports programs to support students with ADHD.
  o Integrate AI-driven monitoring tools to provide real-time feedback and optimize individualized training plans in each classroom.
  o Combining exercise-based interventions with digital platforms (e.g., exergaming, virtual reality, and neurofeedback) may enhance engagement and therapeutic outcomes.
  o Modified physical education (PE) programs should emphasize activities that enhance executive function, cognitive flexibility, and impulse control, such as martial arts, dance, and team sports [127].
  o Collaboration between educators, psychologists, and parents is essential to ensure consistency in PA engagement across different environments.
  o Integration of physical activity with digital therapies in schools, ensuring these hybrid interventions are both accessible and adaptable for diverse ADHD populations.

### 9.2. Individualized Exercise Recommendations for ADHD Subtypes

Not all forms of PA have the same impact on ADHD symptoms. Individualized approaches based on ADHD subtypes and symptom severity can enhance effectiveness [128]:

- Predominantly inattentive subtype:
  o Aerobic activities (e.g., swimming, running, cycling) to improve sustained attention and cognitive processing speed.
- Predominantly hyperactive-impulsive subtype:
  o High-intensity interval training (HIIT) and structured sports (e.g., martial arts, gymnastics) to regulate impulsivity and motor restlessness.
- Combined subtype:
  o A mix of aerobic and coordinative exercises (e.g., dance, team sports) to improve cognitive flexibility, attention control, and emotional regulation.

### 9.3. Enhancing Long-Term Adherence to Physical Activity

For individuals with ADHD, maintaining regular PA routines can be challenging due to difficulties with motivation, executive function, and consistency. Strategies to promote long-term engagement include the following:

- Using gamification and digital tools (e.g., exergaming, virtual coaching) to increase motivation.
- Incorporating social support by engaging in group exercise or team sports.
- Setting personalized goals and tracking progress to reinforce positive behavior changes.
- Adapting exercise routines to lifestyle constraints, ensuring accessibility and sustainability.

By systematically integrating PA into ADHD treatment frameworks, individuals with ADHD can experience enhanced cognitive function, improved emotional regulation, and greater overall well-being.

## 10. Conclusions

ADHD is a multifaceted neurodevelopmental disorder that affects cognitive, emotional, and social functioning across the lifespan. While traditional treatment approaches, such as medication and behavioral therapy, remain foundational, physical activity has emerged as a critical complementary intervention for managing ADHD symptoms. This review highlights that physical activity positively influences core ADHD symptoms, including inattention, hyperactivity, and impulsivity, by enhancing executive function, working memory, and cognitive flexibility. Neurobiological evidence supports the role of physical activity in ADHD management, with studies indicating improvements in dopamine regulation, neuroplasticity, and prefrontal cortex activity. Physical activity interventions should be tailored based on ADHD subtypes, developmental stages, and individual preferences to optimize long-term adherence and effectiveness. Digital innovations, such as exergaming, wearable technology, and AI-driven physical activity programs, present new opportunities for integrating exercise into ADHD treatment strategies. However, a hybrid model—using digital tools to support physical activity—can maximize benefits.

## Figures and Tables

**Table 1:** Studies on sustained PA engagement and improvements with ADHD symptoms using digital or non-digital approaches.

Author	Title	Objective	Results	PA Protocol	Type
Schoenfelder et al. (2017) [124]	Piloting a mobile health intervention to increase physical activity for adolescents with ADHD	To evaluate feasibility and acceptability of an innovative intervention incorporating an mHealth-linked wearable activity tracker (Fitbit Flex) and a Facebook group to increase PA among adolescents with ADHD	Adherence with wearing the Fitbit was high. Adolescents and parents completed the majority of the digital questionnaires, indicating that it is feasible for both teens and parents to digitally track symptoms in real-time from their phones	Step count goals with mobile health (mHealth) technology and social media (FitBit and Facebook)	Digital
Smith A et al. (2013) [75]	Pilot Physical Activity Intervention Reduces Severity of ADHD Symptoms in Young Children	To pilot a before-school physical activity intervention for reducing ADHD symptoms in young children	Sustained involvement in structured physical activity may offer benefits to motor, cognitive, social, and behavioral functioning in young people exhibiting ADHD symptoms.	26 min of continuous moderate-to-vigorous physical activity daily over eight school weeks	Non-digital
Jeyanthi S. et al. (2021) [125]	Effectiveness of structured exercises on motor skills, physical fitness and attention in children with ADHD compared to typically developing children-A pilot study	To evaluate the benefits of a structured, school-based exercise program on motor skill, physical fitness and attention in children with ADHD	Significant improvements in physical fitness, motor skills, and attention were observed in ADHD children compared to the typical developing children	Structured exercise program which included aerobics, resistance exercises, motor skills and attention training 45 min per session, over a period of six weeks for a total of eighteen sessions.	Non-digital
Tsai Y, et al. (2021) [126]	Dose-Response Effects of Acute Aerobic Exercise Intensity on Inhibitory Control in Children With Attention Deficit/Hyperactivity Disorder	To examine whether the effect of acute aerobic exercise on inhibitory control of children with attention deficit/hyperactivity disorder (ADHD) is moderated by exercise intensity	Low- and moderate-intensity exercises resulted in shorter reaction times (RTs) relative to vigorous-intensity exercise during the incompatible condition of the flanker task regardless of task congruency	A flanker task with concurrent collection of electroencephalography (EEG) data after three different intensities of treadmill running	Non-digital
Skalidou S. et al. (2023) [127]	Swimming Activity Alleviates the Symptoms of Attention: Deficit Hyperactivity Disorder (ADHD) a Case Report	To investigate the effects of a swimming exercise program on the symptoms of ADHD in an adult with a diagnosis since childhood.	The swimming learning program significantly alleviated the symptoms of inattention and hyperactivity	Three swimming and two dryland training sessions per week. Each session lasted 90 min and was divided into three stages: a 15 min warm-up, 70 min of aquatic exercises, and a 5 min cool-down.	Non-digital
Sabzi A. et al. (2021) [128]	The Effect of Water Treadmill Exercise on Children with Attention Deficit Hyperactivity Disorder	To determine the effectiveness of water treadmill exercises in children with attention deficit hyperactivity disorder.	Exercise interventions with Water treadmill for eight weeks effectively reduce the symptoms of attention deficit hyperactivity disorder in children and can be used as an appropriate intervention	8 weeks and 3 sessions per week (24 sessions in total), and each session lasted for 30 min. The exercise included running on an Aqua treadmill, the intensity of which was 10 min with 45–40% and 20 min with 55–65% of the maximum heart rate reserve	Non-digital
Zhao L. et al. (2024) [129]	A Digital Cognitive-Physical Intervention for Attention-Deficit/Hyperactivity Disorder: Randomized Controlled Trial	To determine whether BrainFit, a novel digital intervention combining gamified cognitive and exercise training, is efficacious in reducing ADHD symptoms	The BrainFit intervention group had a significantly larger improvement in total ADHD symptoms as compared to those in the control group	BrainFit 12 30-min sessions delivered on an iPad over 4 weeks with 3 sessions per week	Digital
Kollins S. et al. (2020) [130]	A novel digital intervention for actively reducing severity of paediatric ADHD (STARS-ADHD): a randomised controlled trial	To assess whether AKL-T01 improved attentional performance in paediatric patients with ADHD.	The active intervention AKL-T01 significantly improved performance on the primary outcome measure—an objective measure of attention (TOVA API) in paediatric patients with ADHD compared with the control condition	Patients were instructed to use AKL-T01 or the control at home for 5 sessions per day (total time on task about 25 min), 5 days per week, for 4 weeks or the control for 25 min per day, 5 days per week, for 4 weeks.	Digital

## Data Availability

Data are contained within the article.
